# Inherited metabolic disorders: presentation, clinical types, laboratory diagnosis and genetic markers

**DOI:** 10.1186/s13023-025-03979-8

**Published:** 2025-08-11

**Authors:** Aamir Ijaz, Seyyedha Abbas, Maria Shabbir, Yasmin Badshah, Fizzah Abid, Tayyaba Afsar, Suhail Razak

**Affiliations:** 1 NUST School of Health Sciences, National University of Science and Technology (NUST), Islamabad, Pakistan; 2 Department of Biomedicine, Atta-ur-Rahman School of Applied Biosciences (ASAB), National University of Sciences and Technology (NUST), Islamabad, Pakistan; 3 Department of Community Health Sciences, College of Applied Medical Sciences, King Saud University, Riyadh, Saudi Arabia

**Keywords:** Inherited metabolic disorders, Genetic disease, Metabolic pathways, Enzyme defects

## Abstract

Inherited metabolic disorders (IMDs) are classified under rare genetic diseases almost always presenting in newborn and infants. IMDs are classified according to the clinical presentation, diagnosis and prognosis. Several factors are involved in the IMDs pathogenesis. Moreover, almost all IMDs follows the autosomal recessive inheritance pattern. At the basis of these diseases lie genetic mutations that affect metabolic pathways. The diagnosis is made by clinical manifestations in addition to biochemical tests and genetic analysis. Due to the different metabolic pathways involved, the multi-omics approaches can significantly increase diagnosis sensitivity. Early identification and diagnosis of IMD are critical to avoid death or neurological defects. In developing countries, lack of timely diagnosis exists mostly due to socioeconomic factors and unawareness. Research needs to be conducted to find better options for the treatment of IMDs. Understanding the molecular mechanisms of IMDs would be helpful in understanding the challenges that exist for the treatment of IMDs. This review aims to provide understanding regarding the pathogenesis of IMDs. Also, to highlight the challenges that exist in the effective treatment and diagnosis of IMDs.

## Introduction

IMDs are caused by the lack of enzymes or their co-factors that cause errors in metabolic processes. The buildup of toxic substances causes numerous clinical conditions, some of which can be fatal [1]. The International Classification of Inherited Metabolic Disorders (ICIMD) lists 1450 diseases [2]. These disorders affect the body’s ability to process amino acids, organic acids, carbohydrates, and fatty acids. Enzyme defects can result in the accumulation of toxic substances. Moreover, because of enzyme deficiency, the normal metabolic processes are directly impacted. It is known that IMDs should be suspected in newborns, infants or children [3, 4]. The conditions require urgent management and delays in diagnosis or the treatment can lead to death [5]. Early identification using biochemical and molecular biology testing can be valuable [6].

Diagnosis of IMDs in adults is complex because the symptoms may be similar to other diseases. A clinical investigation of 27,702 patients revealed that the utilization of ES/GS made it possible to diagnose 11% of adults with neurological disorders of unknown etiology. Neurological signs are seen in most adults with IMDs, and the average time to a diagnosis after symptom onset is about 15 years. In 40% of IMDs cases, metabolic abnormalities are found [7]. However, the studies indicate the rate of IMDs range from 0.8 to 0.9% when live birth cases were studied [8]. New advancements in detection techniques have further indicated that the prevalence is higher than normal [9]. The global overall IMDs rate is around 50.9 per 100,000 live births, with the highest rate observed in the Eastern Mediterranean region, 75.7 per 100 000 [10]. In developing countries, 33% of all deaths are reported because of IMD, while in children, 0.4% deaths are studied. A 2018 cross-sectional study conducted in India gave a prevalence rate of 3% of IMDs.

Past data indicates that 1 in 1000 newborns were affected with IMDs because of cousin marriages [11]. Organic acidemias have been found to be the most frequent IMDs in children as revealed by different researchers from Pakistan [12, 13]. The diagnosis of IMDs, however, poses a major problem given the current research constraints; therefore, a multi-disciplinary approach is recommended [12]. A survey conducted by Hafeez et al. in 2018 confirmed that non-ketotic hyperglycinemia was the most prevalent disease [14]. Out of 239 patients, 19 various types of inherited metabolic diseases were diagnosed in 180 patients. There were 92 children with carbohydrate metabolism disorders (51%), 59 with lipid storage diseases (32.7%), 18 with organic acidemias and energy defects (10%), and six with amino acid disorders (3.3%); the remaining four (2.2%) had other disorders. From the patients, 58 (32.2%) developed an acute metabolic crisis, 28 (15.5%) developed early onset liver failure, and 24 (13.3%) had mental retardation [15].

## Clinical types of IMDS

Saudubray et al. 2001 have classified IMDs according to the clinical presentation, diagnosis and prognosis. This classification has replaced the previous biochemical classification of IMDs e.g. disorders of carbohydrates, amino acids and lipids etc [16]. IMDs have been mainly classified into three groups.

- *Group 1 (Intoxication Type)*: These disorders include inherited metabolic disorders (IMDs) that cause acute or progressive intoxication due to the accumulation of toxic substances. Examples include aminoacidopathies like maple syrup urine disease (MSUD), phenylketonuria (PKU), urea cycle disorders, organic acidemias, methylmalonic acidemia, tyrosinemia, propionic acidemia, isovaleric acidemia, and galactosemia. Defects in the amino acid metabolic pathways lead to amino acid diseases. When amino acids accumulate in the blood, they lead to amino acidemias, and when they are abnormally excreted in urine, they cause amino acidurias [17, 18]. Symptoms occur due to the accumulation of substances that cannot be metabolized. Conditions like phenylketonuria and maple syrup urine disease fall under this category. These conditions frequently manifest in infants who appear healthy at birth but show symptoms (such as poor feeding or lethargy) after consuming protein. If neglected, symptoms can develop to coma, encephalopathy, or even death [19]. Regression or developmental delays may be seen in older children. Tandem mass spectrometry can be used for newborn screening to identify a variety of amino acid irregularities. Metabolic acidosis, hyperammonemia, hypoglycemia (with or without ketosis), liver failure, and reducing chemicals in the urine are among the biochemical results that are frequently observed [3, 20]. Urine organic acid analysis, enzyme tests, and quantitative plasma amino acid measurements are used to confirm the diagnosis [3].

Organic acidemias or organic acidurias, these conditions involve the accumulation of abnormal (and typically toxic) organic acids, which are excreted in excess in the urine. Various disorders can cause abnormal urine organic acid profiles, including phenylketonuria, maple syrup urine disease, organic acidemias, disorders of ketogenesis, fatty acid oxidation defects, and mitochondrial disorders. Propionic acidemia and Methylmalonic acidemia are examples. Most organic acidemias are present in the newborn period or early infancy [21]. Diagnosis is confirmed by measuring urine organic acids, plasma amino acids, and potentially acylcarnitine profiles and enzyme assays [22].

b. *Group 2: Energy metabolism disorders*: These disorders are characterized by energy utilization deficiency in organs like liver, heart, skeletal muscle or brain. Some disorders in this group may also lead to accumulation of toxic compounds. These disorders can be treated by timely replenishment of energy products e.g. high concentration of glucose. Examples include hypoglycemic disorders, defects in gluconeogenesis, glycogenolysis, hyperinsulinism, pyruvate carboxylase deficiency, krebs cycle defects, fatty acid oxidation disorders, congenital lactic acidemias, and mitochondrial respiratory chain defects [16]. The hypoglycaemia found in these disorders is accompanied by ketosis except fatty acid oxidation defects, which are not accompanied by ketosis. So, these disorders if not monitored properly can cause fatal neurogenic energy crisis as brain can use ketosis if glucose is not available.
c. *Group 3: Complex molecules metabolism disorders* include conditions that affect the synthesis or breakdown of complex molecules. Examples include lysosomal storage disorders such as sphingolipidoses, mucopolysaccharidoses, and glycoproteinoses; disorders of lysosomal enzyme transport; peroxisomal disorders; inherited disorders related with cholesterol synthesis and Golgi apparatus disorders [16] (Table 1). These disorders are progressive, degenerative and are not usually amenable to treatment. None of these disorders is included in newborn screening programmes.

**Table 1:** Overview of IMD clinical classification

Groups	Description	Example
Group 1: Amino acidopathies	Amino acid disorders caused by defects in metabolic pathways, leading to abnormal accumulation or excretion of amino acids.	PKU, MSUD, Urea cycle disorders
Group 1: Organic Acidemias	Characterized by accumulation of toxic organic acid metabolites and increased excretion of organic acids.	Methylmalonic acidemia, Propionic acidemia
Group 2: Energy metabolism disorders	Caused by deficiencies in energy production or utilization, affecting the liver, myocardium, skeletal muscle, or brain	Glycogenolysis defects, Fatty acid oxidation defects, Mitochondrial respiratory chain defects
Group 3: Complex molecules metabolism disorders	Involves defects in the synthesis or catabolism of complex molecules	Lysosomal storage disorders, Peroxisomal disorders, Inherited disorders of cholesterol synthesis

### Genetic defects and effected enzymes in inherited metabolic disorders

Determining the pathogenesis of IMDs is complex. In most cases, lack of a single enzyme can disrupt the metabolic processes. Amino acid disorders (AADs) are caused by a lack of enzymes that breakdown or synthesizes amino acids. Among them, phenylketonuria (PKU), maple syrup urine disease (MSUD), and homocystinuria are most frequently reported [23]. Defficiency of phenylalanine hydroxylase and high phenylalanine levels leads to PKU that cause intellectual disability if left untreated. PKU can be diagnosed when the phenylalanine levels are high in the newborns. These disorders are mostly caused by genes that translate enzymes important to these metabolic processes. In PKU, the PAH gene is mutated that affects the expression and function of phenylalanine hydroxylase [24]. MSUD is caused by mutations of the genes encoding enzymes involved in the breakdown of branched-chain amino acids which result in neurological problems [25].

Other inherited metabolic disorders include organic acidemias: propionic acidemia and methylmalonic acidemia, which are caused by a defect in amino acid, lipid or carbohydrate metabolism leading to accumulation of organic acids in body fluids. propionic acidemia mainly results from the mutations in genes PCCA and PCCB, which encode for components of the propionyl-CoA carboxylase enzyme. The extent of the disease spread depends on how these mutations influence the enzyme’s activity [26].

Congenital carbohydrate metabolism disorders, include glycogen storage diseases (GSDs) and disorders of fructose and galactose metabolism. GSD type I (von Gierke disease) results from enzyme abnormalities that impair glycogen degradation mechanisms, therefore causing it to accumulate in the liver and muscles among other organs. Genetic alterations in enzymes including glucose-6-phosphatase lead to GSD. This enzyme is involved in both glycogen catabolism and gluconeogenesis. The lack of it leads to hypoglycemia, lactic acidosis and liver enlargement. Molecular diagnostic testing for G6PC gene mutation is essential in diagnosis [27]. Moreover, Fatty acid oxidation disorders (FAODs) such as medium-chain acyl-CoA dehydrogenase deficiency (MCADD) impair the degradation of fatty acids for energy production mainly during fasting or during a disease [28]. In MCADD, there are alterations in the gene, ACADM, that encodes the enzyme that breaks down medium-chain fatty acids to produce energy. Moreover, Galactosemia is also caused if carbohydrate metabolism is impacted. Galactosemia is an autosomal recessive disorder that results from deficiency of the enzyme galactose-1-phosphate uridylyltransferase (GALT) that catalyzes the conversion of galactose to glucose. Failure to metabolize galactose leads to the buildup of toxic levels of galactose-1-phosphate, which causes damage to the liver and leads to cataracts and intellectual disability. This disorder is a good example of how carbohydrate metabolism is important in avoiding toxic substrate buildup [29, 30].

Also, lack of pyruvate dehydrogenase complex (PDC) results in energy metabolism disorder and lactic acidosis, delayed development, and neurological disorders [31]. This disorder shows the important interrelationship between glycolysis and mitochondrial energy metabolism. FAODs are a group of inherited metabolic diseases resulting from the defects in enzymes that catalyze the oxidation of fatty acids in the mitochondria. The mitochondrial fatty acid binding protein (FABP) is involved in the fatty acid β-oxidation pathway, which is pivotal in energy metabolism, especially during periods of fasting or periods of increased energy expenditure, such as during exercise. When this pathway is affected, it leads to the accumulation of fatty acid intermediates, which can be toxic to various organs [32]. The deficiency of 2,4-Dienoyl-CoA reductase (DERED), which catalyzes the reduction of 2,4-dienoyl-CoA, results in the buildup of unsaturated fatty acids [33]. Moreover, a concrete example of lipid metabolism disorder is Gaucher disease, which results from the deficiency of glucocerebrosidase, which is involved in metabolism of glycolipids. Gaucher disease is also a type of lysosomal storage disease. The deposition of glycolipids in macrophages results in hepatosplenomegaly, bone disease and neurological manifestations in severe diseases [34].

Furthermore, IMDs are classified as a group of diseases that occur due to mutations in mitochondrial DNA or nuclear genes, which encode for mitochondrial proteins. These disorders target the electron transport chain and cause disease such as leigh syndrome and mitochondrial myopathy. Mitochondrial diseases cause energy impairment in cells, thus resulting in progressive neuronal and muscular dysfunctioning [35] (Table 2).

**Table 2:** Mutated genes that affect the expression and function of translated enzymes that lead to different diseases

Mutated Genes	Translated Enzymes	Diseases
PAH	Phenylalanine hydroxylase	Phenylketonuria (PKU)
BCKDHA/BCKDHB/DBT	Branched-chain alpha-ketoacid dehydrogenase	Maple Syrup Urine Disease (MSUD)
PCCA/PCCB	Propionyl-CoA carboxylase	Propionic Acidemia
G6PC	Glucose-6-phosphatase	Glycogen Storage Disease Type I (von Gierke)
ACADM	Medium-chain acyl-CoA dehydrogenase	Medium-Chain Acyl-CoA Dehydrogenase Deficiency (MCADD)
GALT	Galactose-1-phosphate uridylyltransferase	Galactosemia
PDHA1	Pyruvate dehydrogenase complex	Energy Metabolism Disorder
FABP	Fatty acid binding protein	Fatty Acid Oxidation Disorders (FAODs)
DERED	2,4-Dienoyl-CoA reductase	DERED Deficiency
GBA	Glucocerebrosidase	Gaucher Disease

### Location and mutation types important inherited metabolic disorders

Moreover, for IMDs, both alleles of a gene have to be mutated for the disease to express. Individuals with one mutated gene are asymptomatic but carriers of the disorder, and if both are the genes are mutated, their children have a high risk of inheriting the condition [8]. Some of these are phenylketonuria (PKU), glycogen storage diseases and organic acidemias. Certain IMDs are associated with mutations on the X chromosome, which is especially dangerous for men who have only one X chromosome. Women can also be asymptomatic carriers. An example of an X-linked IMD is ornithine transcarbamylase (OTC) deficiency, a disorder of the urea cycle. Some mutations abolish the activity of enzymes and lead to the severe and early disease manifestations, other mutations cause partial loss of enzyme activity and result in milder or late-onset disease [36].

- *Galactosaemia*: Three well-known inherited metabolic diseases (IMDs), pertaining to the metabolism of galactose are grouped together under the term galactosemia, the most serious of which is classic galactosemia. The underlying cause of this condition is a deficit in the enzyme galactose-1-phosphate uridyltransferase (GALT), which has the potential to cause long-term, multi-systemic involvement and potentially fatal consequences. There are two types of uridine diphosphate galactose 4-epimerase (GALE) deficit: a more common partial deficiency that is usually mild and a rare severe deficiency that resembles classic galactosemia [37]. Furthermore, even though it is incredibly uncommon, a defect in galactokinase (GALK) can result in cataracts alone without any other signs of galactose intolerance [38]. Children having a mutation of Q188R in the GALT gene suffer from a disease called galactosemia. These babies are born with normal weight but they soon begin to lose weight and when they start taking milk, they rarely get back to their birth weight [39]. More than 167 variants of the GALT gene, mapped on chromosome 9, have been described [40, 41]. The Q188R mutation is associated with adverse health consequences where both copies of the gene have this mutation [42, 43]. However, if a baby has one severe Q188R mutation with partial enzyme deficiency, as in the Duarte variation due to N314D mutation, the condition is usually less severe or asymptomatic [38, 44].
- *Persistent Hyperinsulinaemic Hypoglycaemia of Infancy (PHHI)*: It is estimated to occur in about 1/50,000 live born infants, but in Saudi Arabia where consanguineous marriages are frequent, the disease affects 1/2,500 live born infants [45]. PHHI has two forms: one that is spread throughout the pancreas (diffuse) and the other that is localized to a particular area within the pancreas. The localized form is related to the absence of the mother gene in some cells of the pancreas and mutations of genes that regulate insulin. These mutations sit on chromosome 11 and are inherited from the father most of the time [46]. The diffuse form is more serious and results from mutations in these same genes and can make treatment challenging [47].
- *Multiple Carboxylase Deficiency (MCD)*: It is another inherited metabolic disorder that affects the ability of the body to metabolise biotin, an essential vitamin. This happens because of problems with two enzymes: Biotinidase and holocarboxylase synthetase (HCS). If these enzymes are absent, the body cannot metabolize or recycle biotin leading to severe metabolic acidosis and neurological complications [48]. If HCS is absent, the body can not bind biotin to some enzymes and if biotinidase is lacking, the body can not recycle biotin [49]. The biotinidase gene is on chromosome 3, and there have been more than 79 identified mutations [50].
- *Non-Ketotic Hyperglycinemia (NKH)*: It is a genetic disorder that arises from mutations of the AMT, GCSH, and GLDC genes. The disorder is inherited in an autosomal recessive manner, so a child must inherit a defective gene from both parents in order to develop the condition. About 10% of the faulty AMT genes come from three common mutations: IVS 7-1G > A, R320H, and 296 H [51]. Over fifty different mutations have been identified in the GLDC gene and most of these are private mutations [52]. There are some specific mutations, such as R515S, A389V, T269M, and S564I, of which R515S, A389V, and T269M have been reported in several patients in Europe and Canada, and S564I has been identified in the Finnish population [53]. To date, only one mutation in the GCSH gene has been elucidated [54].
- *Hyperphenylalaninemia (HPA)*: It is another heritable metabolic disease resulting from the mutations of genes that synthesize an enzyme known as phenylalanine hydroxylase, which is located in the liver. This enzyme is useful in the degradation of the amino acid phenylalanine. In its severe form called phenylketonuria (PKU), the lack of PAH enzyme function may lead to brain damage if left untreated. Depending on the degree of the PAH deficiency, one may or may not require treatment. When there are additional disorders of pterin metabolism, both HPA and brain chemical balance are impacted and these conditions are usually managed with both tetrahydrobiopterin and neurotransmitter reinforcements. The PAH gene is mapped on chromosome 12, and PAH deficiency is an autosomal recessive disorder. Over 480 variations have been found in this gene, and those with PAH deficiency almost always have two different alterations. Some mutations occur frequently in certain individuals in the society. For instance, the R408W mutation accounts for 30% of PKU incidence in Europeans and R243Q among 13% of oriental PKU patients [55, 56]. The specific genetic mutations correlate well with the biochemical levels of phenylalanine in the blood before treatment and how much phenylalanine a person can tolerate, but other factors affect the clinical symptoms [55].
- *Urea cycle disorders*: These are inherited metabolic diseases that result from the deficiency of enzymes that metabolise toxic nitrogen. These enzymes are OTC, arginase, Carbamoyl Phosphate Synthetase (CPS), Argininosuccinate Synthetase (ASS), Argininosuccinate Lyase (ASL), and N-Acetylglutamate Synthase (NAGS). Abnormalities with these enzymes result in high levels of ammonia in the blood which is poisonous. OTC deficiency is the most prevalent form of the urea cycle disorder and is inherited in an X-linked recessive pattern, which affects mostly males. OTC deficiency can be diagnosed by genetic testing to find out whether a mother has the gene or not [57, 58].
- *Branched-chain organic acidurias, or organic acidemias*: These disorders arise when the body is unable to metabolize branched chain amino acids (BCAAs), some amino acids that are branched in their structure. The common disorders of this kind are isovaleric aciduria, methylmalonic aciduria, propionic aciduria, and maple syrup urine disease. MSUD is a genetic disorder that is caused by over 150 mutations in three genes; E1α, E1β, and E2 and is a condition that prevents the body from metabolizing some amino acids properly, and this leads to mild to severe symptoms [59]. Another condition is called IVA, but it is another condition that has a great variability of its manifestation. It is hard to see how one links the specific genetic mutations to the symptoms that people develop. However, children with the 932 C > T mutation, who were identified through newborn screening, have a milder form of IVA and may be asymptomatic [60].
- *Propionic aciduria (PA)*: It is a genetic disorder caused by mutations in two genes: PCCA and PCCB. These genes are involved in making parts of an enzyme that is used in the body to metabolise specific fats and proteins. Over 50 mutations in PCCB gene and over 30 in the PCCA gene have been reported [61]. Knowing the relationship between the genetic mutations of a person and the intensity of the condition will enable the physician to anticipate the disease’s progression and offer improved and more appropriate treatment options to the patient [62].
- *Methylmalonic aciduria (MMA)*: It is an autosomal recessive disorder that results from a deficiency of methylmalonyl-CoA mutase (MCM) that is coded for by the MUT gene. This enzyme plays a role in the digestion of certain fats and proteins and when it is not working as it should, toxic compounds accumulate in the body. MMA can also arise from defects in synthesizing a required helper molecule known as 5-deoxyadenosylcobalamin (AdoCbl), which is linked to vitamin B12. Over 50 mutations have been reported in individuals with MMA resulting from dysfunction of this enzyme [63]. Further, researchers have identified two other genes, MMAA and MMAB, which are also implicated in vitamin B12 metabolism but have been found to carry pathogenic mutations. The MMAB gene codes for an enzyme that assists in the activation of vitamin B12 and the MMAA gene is believed to aid in the transport of vitamin B12 into the mitochondrion, the cellular organelle responsible for energy production [64, 65].
- *Tyrosinemia*: Different genetic disorders are associated with the metabolism of tyrosine, an amino acid that is catabolized by this pathway. These conditions are grouped under inherited metabolic disorders (IMD): Hereditary tyrosinemia type I is characterized by rickets, liver disease and renal failure. This condition is brought about by more than 100 mutations in the FAH gene [66]. Of all the mutations, IVS12 + 5(g-a) is found in 25% of all patients worldwide, but it is found in more than 90% of French Canadians [67]. Richner Hanhart syndrome or hereditary tyrosinemia type II results in formation of blisters on the palms of the hand and soles of the feet as well as eye problems such as keratitis. There may be no symptoms in hereditary tyrosinemia type III or sometimes mental retardation. Alkaptonuria is another inherited disorder of tyrosine metabolism that results in osteoarthritis among adults [68, 69].
- *Glycogen Storage Disease (GSD)*: These diseases fall under IMD category wherein the body fails to metabolize glycogen, a stored sugar. GSD types Ia and Ib are due to deficiencies of enzymes that metabolize glucose. In type Ia, the deficiency is of the enzyme glucose-6-phosphatase while in type Ib, the deficiency is of glucose-6-phosphate translocase. Since the identification of the G6PC gene on chromosome 17 in 1993, more than 75 mutations have been identified to cause GSD Ia [70, 71]. Furthermore, over 186 mutations have been described of the gene which encodes G6PT, a protein involved in glucose-6-phosphate transport [72]. The diagnosis of GSD is clinical and biochemical with genetic tests identifying the mutations [71] (Table 3).

**Table 3:** Genetic causes of important inherited metabolic disorders

Disorders	Genetic causes
Galactosemia	Mutations on GALT gene, chromosome 9, 167. Q188R mutation widely studied.
Persistent Hyperinsulinaemic Hypoglycaemia of Infancy	Mutations in SUR1 or Kir6.2 genes on chromosome 11p15.
Multiple Carboxylase Deficiency	HCS on chromosome 21q22.1 and biotinidase on chromosome 3p25, 79 mutations identified
Non-Ketotic Hyperglycinemia	AMT, GLDC, and GCSH genes, around 50 GLDC mutations identified
Hyperphenylalaninemia	PAH gene on chromosome 12, 500 mutations described.
Urea Cycle Disorders	Mutations in urea cycle enzymes, especially OTC gene (X-linked)
Branched-Chain Organic Acidurias/Acidemias” cell	Mutations in E1α, E1β, E2 genes (MSUD), IVD gene (IVA), PCCA or PCCB genes (PA), MUT locus (MMA)
Tyrosinemias	FAH gene on chromosome 15q23–25, > 40 mutations described.
Glycogen Storage Disease	G6PC gene on chromosome 17q21, G6PT on chromosome 11q23, over 75 and 65 mutations described, respectively

### The laboratory evaluation of IMDs

The management of IMDs is systematic, and starts with simple blood tests and then progresses to more complicated tests that help in identification of the particular disorder [73].

A diagnostic scoring system named Rawalpindi Inherited Metabolic Diseases Score (RISc) has also been devised and tested for its diagnostic accuracy IMD in resources-limited settings (73a).

**Table 4:** Rawalpindi inherited metabolic diseases score (RISc) score card variables for selection of patients to undergo specialized tests (Ref 73a)

Parameter	Score
Consanguinity	2
Affected Sibling (with similar presentation)	2
Failure to thrive	1
Jaundice	0.5
Convulsions	1
Raised Plasma Ammonia (> 150 µmol/L)	1
Urine for presence of non-reducing substance	1
Altered Acid Base Status	0.5
Raised Plasma Lactate	0.5
Deranged LFTs	0.5
**Total Score**	**10**

Table 4 shows the parameters and scores used for calculation of RISc. It comprises clinical findings and routine laboratory investigations to select babies who are required to undergo sophisticated tests for diagnosis of a particular disorder. A patients with low RISc (0.5–2.5) do not need further investigations while medium RISc (3.0-5.5) and high RISc [6–10] require further investigations depending on available resources.

To diagnose an IMD, doctors search for raised levels of biochemicals in the blood, urine or CSF. They may also run assays on enzyme in skin, RBC, liver or muscle tissue, and occasionally on a individual’s DNA. When doctors have any indication that a child has an IMD, the first diagnostic test that is carried out is a complete blood count (CBC). This test determines if the IMD is impacting any of the various types of blood cells. The CBC can also show that a child has an infection or sepsis that could result in a dangerous metabolic crisis [74].

- *Plasma Glucose*: Some IMDs can precipitate hypoglycemia or low blood sugar levels and these include: Medium-chain acyl CoA dehydrogenase deficiency, Glycogen storage diseases, Gluconeogenic disorders, and Genetic fructose intolerance [75]. These disorders concern the inability to break down fatty acids [76]. Other conditions that may lead to hypoglycemia include; organic acidemias, mitochondrial diseases, and amino acid disorders. Metabolic acidosis is a common feature in patients with Organic acidemias and can be diagnosed by blood gas analysis [77]. If the quantity of ammonia in the blood is high (hyperammonemia), then the plasma ammonia test can be used to diagnose it. This test is done by collecting blood samples in a special tube containing lithium heparin. Ammonia > 300 µmol/L is toxic to the brain and is seen in UCAs and some Organic acidemias, which needs emergent management [78]. If there is elevated ammonia, further tests are needed to identify the cause of hyperammonemia and these are liver function tests, urine organic acid analysis, and plasma amino acid analysis.
- *Lactic acid testing* in blood can reveal different metabolic disorders, including disorders of oxidative metabolism, mitochondrial disorders, defects in sugar and fat breakdown (gluconeogenic disorders, glycogen storage diseases, organic acidemias). This assists in screening for a situation known as lactic acidosis which is a situation whereby there is excessive accumulation of lactic acid in the blood stream [79].
- *Liver function tests* may be normal, they should still be performed to exclude liver issues, particularly in the presence of hyperammonemia [80].
- *Plasma electrolytes* such as high potassium levels, also known as hyperkalemia or low sodium levels, also known as hyponatremia may point to metabolic acidosis or conditions in which the body loses excess amounts of salt [81].
- *Urine Analysis*: Analyzing the urine for some biochemicals can also help diagnose metabolic diseases. For instance, if sugar related substances are detected, then it may suggest diseases such as galactosemia or inherited fructose intolerance, which are characterized by carbohydrate metabolism disorders [82]. In some cases, urine with low pH can be used to diagnose conditions such as metabolic acidosis, or renal tubular acidosis which is a condition whereby the body is unable to maintain the correct balance of acids and alkalis [83].

Hypoglycemia, hyperammonemia, blood problems, liver failure, and kidney disease, if present along with unexplained acid-base disturbances, increase the likelihood of an IMD [84]. These disorders are less recognized in the developing countries such as Pakistan because of certain socio-economic barriers, hence more people in such countries suffer from these disorders and die from them. The early diagnosis can greatly decrease the chance of developing a long-term neurological disorder [85]. But, diagnosing these disorders may be complicated if the signs are obscure or in case of other diseases such as infections or birth traumas [5]. The patient’s symptoms and signs should be identified early to help diagnose IMDs, and the patient should be immediately referred to adequate medical facilities for improved results [78]. Also, the diagnosis of these disorders can be quite problematic because of the problems with sampling and sample preservation [86]. It can also make the process costly since simple biochemical tests have to be carried out before more elaborate tests are done. The screening tests for some specific IMDs include plasma amino acid (AA) testing and urine organic acid (OA) analysis. Plasma AA testing applies ion exchange chromatography (IEC) while urine OA analysis employs the gas chromatography-mass spectrometry (GCMS). These tests aid in determining the specific disorder but in most cases, a more elaborate analysis is conducted through enzyme and genetic analysis [87].

### Important biochemical presentations

#### Metabolic acidosis

For patients with metabolic acidosis, that is, if the blood pH is low and there is no significant production of ketones, doctors have to measure lactate levels. If the level of glucose and Lactate are normal during metabolic acidosis, then it may be a case of Renal Tubular Acidosis (RTA) [79]. Low glucose levels can be as a result of fatty acid oxidation while high levels of lactate may be due to pyruvate dehydrogenase complex, which is involved in energy metabolism.

When metabolic acidosis is associated with ketosis (the presence of ketones), other investigations that should be done are blood glucose, lactate, and ammonia levels. When glucose is high and ammonia either normal or low it can be due to diabetesor ketolytic abnormalities. On the other hand, high Glucose with high ammonia is usually observed in diseases such as branched-chain organic acidurias.In some cases, metabolic acidosis with ketosis may be present in the absence of alterations in lactate and glucose concentrations such as in organic acidurias or late-onset MSUD. Also, if a person has hypoglycemia together with metabolic acidosis and ketosis, the level of lactate should be assessed. If there is high lactate, it may indicate an issue with gluconeogenesis or issues with the respiratory chain which is a part of the cell’s energy production process [6].

#### Hypoglycemia

Low blood sugar is related to several inherited metabolic diseases including fatty acid oxidation disorders, Glycogen storage diseases, Gluconeogenic disorders, Congenital fructose intolerance and Galactosemia [88, 89]. It may also occur in mitochondrial disorders, organic disorders, and disorders of amino acids. There are some diseases which are accompanied by hypoglycemia and hepatomegaly, such as hereditary fructose intolerance, tyrosinemia type I, galactosemia. However, in some of the glycogen storage disease, GSD III and VI, hypoglycemia is accompanied by isolated hepatomegaly, ketosis and fasting hypoglycemia.

When hypoglycemia is present but there is no gross liver enlargement, the following should be looked at metabolic acidosis and ketosis. If high lactate levels (hyperlactatemia) are present, it can point towards conditions such as organic acidurias or late onset Maple Syrup Urine Disease (MSUD) [90]. Regarding fatty acid oxidation (FAO) deficiencies, hyperinsulinism or cortisol insufficiency, it should be said that ketosis or acidosis are missing, which means that ketone bodies or excessive levels of acidity are not detected in the blood under these conditions.

#### Hyperammonemia

This is especially true if a child is vomiting without a known cause, if he or she is lethargic or if the child is showing signs of encephalopathy. Hyperammonemia can be associated with liver disease, FAO defects, organic acidemias, or urea cycle disorders. If hyperammonemia is present together with metabolic acidosis, then it may indicate an issue with the metabolism of pyruvate which plays a role in the mitochondrial respiratory chain [91].

#### Hyperlactatemia

In order to diagnose IMDs, other conditions that can cause elevated blood lactate levels must be first excluded, which include circulatory failure, anoxic injury, infection, or abnormalities in oxygen utilization in cells. These conditions can all raise the lactate level. Oxidative metabolism is found to be disturbed in glycogen storage diseases, lactic acidosis, pyruvate metabolism disorders and the mitochondrial disorder and diseases related to gluconeogenesis. For example, in glycogenosis type III (also known as glycogen synthase deficiency), signs like an enlarged liver (hepatomegaly) and high lactate levels after eating suggest the diagnosis. If there is high lactate after meals with neurological signs, it may be due to an enzyme PDH or Multiple Carboxylase Deficiency. On the other hand, if high lactate appears after fasting it may be associated with fatty acid oxidation or fructose 1,6-bisphosphatase deficiency or glycogenosis type I [92].

#### Ketosis

Generally, ketosis (that is free of hypoglycemiais or acidosis) is the body’s physiological response to fasting, catabolism, or ketogenic diets [93]. Glycogenosis types I, III, and IX are characterized by ketosis without acidosis, hypoglycemia, and hepatomegaly [94]. MCAD, adrenal diseases, ketotic hypoglycemia, or errors in ketolysis can all result in ketosis without hepatomegaly or hypoglycemia [95].

#### Specialized laboratory testing

In IMD cases, it is important to obtain samples for unique investigations at moments when the symptoms are most manifest. One of the tests is plasma amino acid (AA) analysis, which serves to identify disorders in the metabolism of amino acids. This test requires 2–3 ml of the blood that is taken in a lithium heparin tube and the method used here is ion exchange chromatography (IEC). This method is especially useful in the diagnosis of disorders in amino acid metabolism [14]. Urine amino acid analysis is less useful but can be beneficial in some cases. There are situations, for instance, non-ketotic hyperglycinemia, where determination of cerebrospinal fluid (CSF) amino acid is useful in conjunction with plasma. This test employs 1–2 ml of CSF and if the CSF to plasma glycine ratio is greater than 0.08, then it points to a complication in the metabolism of glycine, hence non-ketotic hyperglycinemia. A urine organic acid analysis is used by doctors to identify disorders such as methylmalonic acidemia, propionic acidemia, and isovaleric acidemia. This test involves application of gas chromatography/mass spectrometry (GC/MS) on a 10–20 ml urine sample to identify these organic acidemias [14].

Another useful test is acylcarnitine profiling that is used to diagnose disorder in fatty acid oxidation (FAO) and some of the organic acidemias. This test is done on dried blood spots or plasma by means of a method known as liquid chromatography/mass spectrometry/mass spectrometry (LC/MS/MS). Sometimes, it is necessary to determine enzyme activity in the tissues such as skin, muscle or liver that can be determined by biopsy [96]. Also, urine glycosaminoglycan and oligosaccharide provide information about lysosomal storage diseases, and serum very long-chain fatty acid is used to identify peroxisomal diseases. These tests offer helpful data in ascertaining the diagnosis of metabolic disorders [97].

#### Molecular genetic testing

Although biochemical testing continues to be the most preferred diagnostic approach in IMDs, the introduction of molecular genetic testing has been gaining relevance owing to its cost effectiveness and enhanced precision [98]. In case of specific genetic conditions such as congenital disorders of glycosylation or mitochondrial energy metabolic defects, where biochemical assays either do not exist or are not easily obtainable, genetic diagnosis is invaluable [99]. Molecular testing is also likely to play a role in the future development of therapies [100]. Revolutionary techniques that have been developed in the recent past, such as whole exome sequencing (WES), are on the rise and are particularly beneficial since they yield faster results and are more affordable, which will probably lead to increased diagnosis of IMDs [7].

### Diagnosis options

Diagnosis of IMDs necessitates a multi-omic view combining clinical evaluations, biochemical studies, further genetic tests and recent cutting-edge omics technologies. Rapid and precise diagnosis is of utmost importance for the appropriate therapy as well. When one becomes suspicious due to the clinical signs, asking for blood and urine tests to see altered metabolite levels helps in understanding if something is defective. Commonly found abnormalities on blood tests include high ammonia, lactic acid and amino acids; analysis of urine organic acids. Often tandem mass spectrometry (MS/MS) is essential to recognize organic acidemias [101]. Early detection programs have become more sophisticated and are moving towards newborn screening. From there, the activity of certain enzymes implicated in a disorder will be assessed through enzyme assays. These are actually quite useful in diagnosis of lysosomal storage diseases.

Moreover, multiomics approaches that incorporate metabolomics, proteomics and transcriptomics also are helpful to identify metabolites causing IMDs. Metabolomics identifies particular trends in metabolic compounds associated with IMDs, while proteomics considers the dysfunctional enzymes and searches for disease mechanisms and therapies. Transcriptomics allows exploring gene expression in response to signals from transcription and revealing metabolic pathways’ dysregulation in IMDs’. Such combined approaches have worthily raised accuracy in the diagnosis [102]. IMDs are primarily diagnosed by laboratory tests that detect abnormal levels of metabolites in the blood, urine, or tissues. For amino acid disorders, high performance liquid chromatography (HPLC) is normally used to quantify amino acid levels in plasma, and for organic acidemias, gas chromatography-mass spectrometry (GC-MS) is used to analyze the urine. Carbohydrate metabolism disorders are usually diagnosed by enzymatic assays or molecular testing for gene mutations. The diagnosis of IMDs has been revolutionized by the advent of molecular characterization [103]. Next-generation sequencing (NGS) allows for the sequencing of multiple genes at one time, which is very helpful in disorders with similar biochemical profiles because the conventional metabolic tests may not be conclusive [104].

### Therapeutic options

Treating IMDs involves diet control, which can be done by either limiting the consumption of toxic metabolites or supplementing insufficient enzymes or cofactors [105]. A rigorous low-phenylalanine diet is required in PKU patients to avoid neurotoxicity. In MSUD, dietary restriction of branched-chain amino acids is the cornerstone of treatment. For instance, depending on the underlying disease, managing organic acidemias could involve limiting protein consumption and supplementing with cofactors like biotin or cobalamin [106]. In particular, gene therapy has demonstrated significant potential for treating several IMDs. New opportunities for correcting genetic mutations have been presented by recent developments in molecular gene-editing technologies, such as the CRISPR-Cas9 system [107]. Subsequent studies using the same vector demonstrated efficacy in correcting GSD type I and some organic acidemias disease, but long-term safety and efficacy is unclear. The substitute therapy called Enzyme replacement therapy (ERT) has been proven to be the successful treatment in a few lysosomal storage disorders, which are a subgroup of IMDs [29]. Still, ERT is not widely applicable to enzyme deficiencies in the mitochondria such as FAODs. Studies are conducted to develop compounds that can increase the activity of residual enzymes or overcome metabolic block for these disorders [108].

## Conclusion

Significant advances have been made in the diagnosis and management of Inherited Metabolic Disorders. These advancements have augmented the diagnostic accuracy. Although traditional diagnostics remain crucial, these are now complemented and considerably broadened by next-generation genetic testing and multiomics analysis. These methods facilitate a more multi-factorial understanding, support the discovery of overlapping symptoms, and allow to delve into the molecular mechanisms of IMDs. Although there has been much progress in the field of IMDs, there continue to be many difficulties in diagnosing and treating these disorders. The main problem is its diagnosis. Since IMDs are so rare, most clinicians would not recognize the symptoms of an IMD that delay the diagnosis and treatment. Another challenge is the heterogeneous presentation of IMDs. Even with the same disorder, the symptoms can be very different, especially with adults. So this variability can cause IMDs to be mistaken by other diseases. Availability of genetic testing, the most important aspect of proper diagnosis, is still lacking in many parts of the world, particularly in low- and middle-income countries. Although genetic testing has become more commonplace, it still is expensive, and not easily accessible for many patients. Speaking of treatment, although the therapies like ERT and gene therapy seem hopeful, they are not without their limitations. These treatments are not only costly, but they may have side effects. More research is definitely needed to find better, cheaper, more readily available treatments. But hopefully, the future of IMD research will involve the incorporation of a multi-omics technology. These technologies allow for novel therapeutic targets as well as a greater understanding of the mechanistic basis of diseases, which in turn will help in treatment and management of IMDs.

## Data Availability

Raw data will be available from the corresponding author on request.
